# Functional selectivity of cardiac preganglionic sympathetic neurones in the rabbit heart^☆^

**DOI:** 10.1016/j.ijcard.2018.03.119

**Published:** 2018-08-01

**Authors:** Reshma A. Chauhan, John Coote, Emily Allen, Pott Pongpaopattanakul, Kieran E. Brack, G. Andre Ng

**Affiliations:** a Department of Cardiovascular Sciences, University of Leicester, UK; b University of Birmingham, UK; c NIHR Leicester Biomedical Research Centre, Leicester, UK; d University Hospitals of Leicester NHS Trust, Leicester, UK

**Keywords:** APD, action potential duration, CL, cycle length, ERP, effective refractory period, LV, left ventricle, LVP, left ventricular pressure, MAP, monophasic action potential, RT, restitution, VA, ventriculo-atrial, VFT, ventricular fibrillation threshold, Sympathetic nervous system, Cardiac electrophysiology, Cardiotopic, Sympathetic chain, Heart

## Abstract

**Background:**

Studies have shown regional and functional selectivity of cardiac postganglionic neurones indicating there might exist a similar heterogeneity in spinal segmental preganglionic neurones, which requires further investigation.

**Methods:**

Right and left sympathetic chains were electrically stimulated from T6 to T1 in the innervated isolated rabbit heart preparation (*n* = 18). Sinus rate, left ventricular pressure, retrograde ventriculo-atrial conduction, monophasic action potential duration, effective refractory period, ventricular fibrillation threshold and electrical restitution were measured.

**Results:**

Right sympathetic stimulation had a greater influence on heart rate (T1-T2: right; 59.9 ± 6.0%, left; 41.1 ± 5.6% *P* < 0.001) and left stimulation had greater effects on left ventricular pressure (T1-T2: right; 20.7 ± 3.2%, left; 40.3 ± 5.4%, *P* < 0.01) and ventriculo-atrial conduction (T1-T2: right; −6.8 ± 1.1%, left; −15.5 ± 0.2%) at all levels, with greater effects at rostral levels (T1-T3). Left sympathetic stimulation caused shorter monophasic action potentials at the base (T4-T5: right; 119.3 ± 2.7 ms, left; 114.7 ± 2.5 ms. *P* < 0.05) and apex (T4-T5: right; 118.8 ± 1.2 ms, left; 114.6 ± 2.6 ms. *P* < 0.05), greater shortening of effective refractory period (T4-T5: right; −3.6 ± 1.3%, left; −7.7 ± 1.8%. *P* < 0.05), a steeper maximum slope of restitution (T4-T5 base: right; 1.3 ± 0.2, left; 1.8 ± 0.2. *P* < 0.01. T4-T5 apex: right; 1.0 ± 0.2, left; 1.6 ± 0.3. *P* < 0.05) and a greater decrease in ventricular fibrillation threshold (T4-T5: right; −22.3 ± 6.8%, left;-39.0 ± 1.7%), with dominant effects at caudal levels (T4-T6).

**Conclusions:**

The preganglionic sympathetic efferent axons show functionally distinct pathways to the heart. The caudal segments (T4-T6) of the left sympathetic chain had a greater potential for arrhythmia generation and hence could pose a target for more focused clinical treatments for impairments in cardiac function.

## Highlights

• Direct evidence that the preganglionic sympathetic efferents show functionally distinct electrophysiological pathways in the rabbit heart
• T1-T3 had a greater influence on heart rate and left ventricular pressure
• T4-T6 of the left sympathetics had greater potential for arrhythmia generation

## 1 Introduction

Excitation of small distal branches of cardiac postganglionic nerve bundles, elicits highly localized changes in spatially restricted portions of the heart [[1], [2], [3]]. Studies reviewed by Randall [4] support this selectivity and led him to suggest the innervation from the spinal cord preganglionic neurones must also be regionally specific. This depends on the extent of the discharge zone and spread of synaptic excitation in groups of target selective postganglionic neurones in the stellate ganglia and ansae subclavia, from where the cardiac postganglionic innervation originates [[5], [6], [7], [8], [9]], and other postganglionic neurones that exist outside of the stellate ganglia. Therefore, to elicit an action in a discrete region of the heart by activation within the spinal cord would require projections of spinal axons onto discrete ganglion cells. Such an organization is supported by studies of innervation and transmission in sympathetic ganglia, which indicate that the discharge of a single ganglion cell is dominated by a single preganglionic fibre [[10], [11], [12]]. Therefore the anatomy and physiology of autonomic ganglia favour the likelihood of separate functionally discrete sympathetic preganglionic efferents synapsing with regionally and functionally discrete ganglion neurones projecting to the heart. So far this has not been demonstrated.

The potential ability of the spinal sympathetic preganglionic neurones to selectively regulate different cardiac regions and functions was previously investigated by chemically exciting these neurones in the spinal cord of rats [9] or by electrical stimulation of left and right upper thoracic ventral roots in dogs [13]. However, a discrete spinal segmental influence on chronotropic or inotropic functions or on selective regional targets was not apparent. In the pioneering studies of Norris et al. [14], a right-left difference was observed and the magnitude of responses varied considerably between roots. In accord, studies on dogs by Kostreva et al. [15] showed marked differences in the contribution of different ventral roots to responses in specific cardiac postganglionic nerves like the ventrolateral cervical cardiac nerve, the ventromedial cervical cardiac nerve and the vagosympathetic trunk, each of which has branches to different regions of the heart. Ardell et al. [16] showed that stimulation of branches of some of these postganglionic nerves had preferential functional cardiac effects. However, there is a lack of evidence for selective functional influences of the sympathetic preganglionic nerves arising from different spinal cord segments, especially of direct evidence for electrophysiological effects and arrhythmia initiation.

In a recent study [17], we reported on the functional heterogeneity of the left and right cardiac sympathetic nerves but this was limited because the stimulus was restricted to the T2-T3 level on the sympathetic chain. This would activate all axons from more caudal segments and miss those projecting from T1-T2. Thus it could not answer the question regarding a selective functional influence.

Therefore, in the present experiments we measured the effects of electrical stimulation of each of left or right sympathetic chains at discrete sites from T6 to T1, on sinus rate, left ventricular pressure, retrograde ventriculo-atrial (VA) conduction, action potential duration (APD) and refractory period, ventricular fibrillation threshold (VFT) and electrical restitution. For this purpose the studies were conducted using our isolated innervated rabbit heart preparation [18].

The results provide evidence for functionally distinct and dominant sympathetic preganglionic pathways to the heart. They also reveal for the first time that activity from caudal spinal segments could potentially be more arrhythmic. We consider that this knowledge improves our understanding of sympathetic nerve control of cardiac performance and could lead to improvements in methods of treatment for impairments in cardiac function.

## 2 Methods

### 2.1 The innervated isolated heart preparation

The innervated isolated heart preparation has been described previously [18]. Briefly, adult male New Zealand white rabbits (1.7–2.5 kg, *n* = 18) were sedated with Medetomidine Hydrochloride (0.2 mg/kg), Ketamine (10 mg/kg), and Butorphanol (0.05 mg/kg) (s.c.) with anesthesia maintained using i.v. Propofol via the ear vein. The trachea was intubated and ventilated, via a small animal ventilator (Harvard Apparatus Ltd., Edenbridge, Kent, UK; O_2_ –air mixture), at 60 breaths per minute. The subclavian vessels were ligated and cut peripherally. Animals were then heparinized (1000 IU, i.v.) and euthanized with an overdose of Euthetal (111 mg/kg, i.v.). Ventilation was switched off and the thoracic cavity exposed by removing the anterior portion of the ribcage. The descending aorta was cannulated and the heart was then flushed with ice-cold Tyrode solution. The pericardium was cut and the mammary arteries were tied off with silk sutures. The vertebral column was then exposed and transected at the 12th thoracic vertebra and 1st cervical vertebra and surrounding tissues were removed.

All procedures conformed to the ethical guidelines in the Animal Scientific Procedure Act 1986 (ASPA), in accordance with Guide for the Care and Use of Laboratory Animals published by the US National Institute of Health (NIH Publication No. 85-23, revised 1985), and followed the criteria of the EU legislation on the protection of animals used for scientific purposes (Directive 2010/63/EU, 2010).

### 2.2 Langendorff perfusion

The heart was perfused with Tyrode solution (37 °C, pH of 7.4 maintained by bubbling with 95% O_2_/5% CO_2_) via the descending aorta, consisting of Na^+^ 138.0, K^+^ 4.0, Ca^2+^ 1.8, Mg^2+^ 1.0, HCO_3_^−^24, H_2_PO_4_^−^0.4, Cl^−^ 124, Glucose 11 (mM) at a flow rate of 100 ml/min via a Gilson minipulse 3 peristaltic pump (Anachem, Luton, UK). The Thebesian venous effluent was drained via the left ventricle using a 1–2 mm wide catheter.

A transducer (MLT0380/D ADInstruments Ltd., Charlgrove, UK) was used to measure the perfusion pressure (PP) and another was attached to a fluid filled latex balloon inserted through the left atrium into the left ventricular cavity to measure the left ventricular pressure (LVP). The balloon was inflated with distilled water to reach an end diastolic pressure of 0–5 mm Hg.

### 2.3 Cardiac electrical recording and pacing

A pacing catheter (ADinstruments Ltd., Chalgrove, UK) was inserted into the right ventricle to deliver a stimulus to the heart at double the diastolic pacing threshold using a constant current stimulator. APD restitution and ventricular fibrillation pacing protocols were used.

Monophasic action potentials (MAPs) were recorded extracellularly from the left ventricular epicardial surface of the heart at the base and apex, by applying two MAP electrodes (Harvard Apparatus Ltd., Holliston, Massachusetts, US. Model number 73–0150), gently onto the surface. Platinum hook electrodes (Grass Instruments, Astro-Med Inc., USA) were attached to the right atria in order the measure the atrial electrogram. Dromotropic effects of left and right sympathetic stimulation at different levels were measured from right atrial electrograms during constant ventricular electrical pacing to obtain VA conduction by measuring the delay from right ventricle pacing spike to the atrial electrogram, as described before [17].

### 2.4 Pacing protocols

#### 2.4.1 Electrical restitution

The restitution (RT) protocol consisted of pacing at a cycle length (CL) of 240 ms for 25 S1 drive train beats, followed by a single extra stimulus (S2). S2 had an initial CL of 240 ms, which was decreased in increments of 10 ms until an S2 CL of 200 ms was reached. It was then decreased in 5 ms increments until the effective refractory period (ERP) was reached. The ERP was defined as the longest S1-S2 interval that failed to capture the S2 beat.

Construction of MAP duration restitution curves by plotting S2 MAP duration vs. diastolic interval (DI = interval between the S1-and S2-MAP signals minus S1-MAPD_90_) allowed arrhythmia susceptibility to be measured from the slope of the curve. MAP duration was identified from time of activation (T_act_) to 90% of repolarization (MAPD_90_) using the programme NewMap (Francis Burton, Glasgow University, UK). Using Microcal Origin (v 6.0, Origin, San Diego, CA, US), an exponential curve was formulated using the following function: MAPD_90_ = maximum MAPD_90_[1 − e^−DI/T^] where T = time constant and the maximum slope of the curve (RT slope) was acquired by measuring the peak value of the first derivative [19].

#### 2.4.2 Ventricular fibrillation threshold

The heart was paced with 25 pulses at 240 ms cycle length followed by burst pacing with 30 pulses with 30 ms cycle length. This was repeated and current was increased from 0.5 mA in 0.5 mA increments until ventricular fibrillation (VF) was induced. The minimum current that induced VF was measured as the VF threshold (VFT).

### 2.5 Sympathetic nerve stimulation

**Fig. 1:**
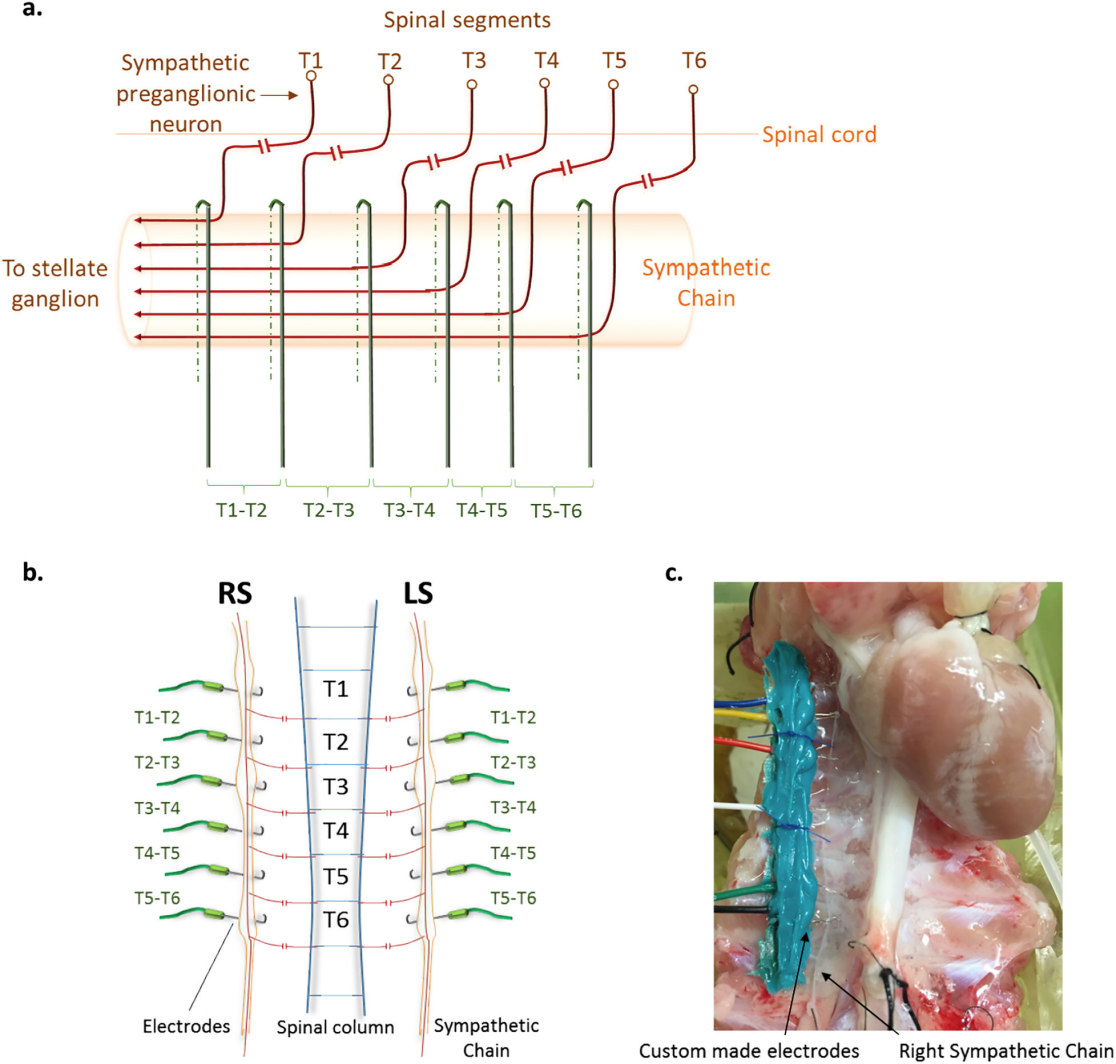
Diagram to illustrate the methods used to stimulate the cardiac sympathetic preganglionic neurones projecting to the stellate ganglion from the upper thoracic spinal cord. (a) Schematic to show the theoretical basis underlining the experimental approach whereby the placement of the stimulating electrodes activates an increasing contribution of sympathetic preganglionic axons from successive spinal cord segments. (b) Drawing showing the placement of electrodes on right and left sympathetic chains parallel to the spinal column at levels between T1-T6. These six electrodes were carried on a narrow plug attached to the posterior chest wall and the whole arrangement was insulated with Kwik-Sil adhesive. (c) Image of dissected right sympathetic chain after placement on custom made electrodes in positions T5-T6, T4-T5, T3-T4, T2-T3 and T1-T2. (b) Image of final preparation with both sympathetic chains positioned on electrodes and embedded with Kwik-Sil adhesive.

The sympathetic chains were identified on either side of midline and parallel to the spinal column. Each chain was dissected free of connective tissue, transected below T6 and decentralized, by sectioning the rami from levels T5-T6 to T1-T2. Custom made electrodes consisting of a strip of 6 electrodes, were positioned between T6-T1 on both the left and right sides. Such an arrangement meant that each successive pair of electrodes, from T5-T6 to T1-T2, stimulated all the preganglionic efferent axons from each preceding segment as well as those at which the electrodes were positioned as shown in Fig. 1. The nerves were lifted gently onto to each electrode and insulated from surrounding tissue and fluid with Kwik-Sil adhesive. The left and right sympathetic chains were stimulated with a train of square wave pulses at 5–7 Hz via pairs of electrodes (cathode proximal to stellate ganglion) at five sites, T5-T6, T4-T5, T3-T4, T2-T3 and T1-T2 at ×2 threshold voltage. The threshold voltage was defined as the lowest voltage that gave an increase in both LVP (of 3–4 mm Hg) and heart rate (of 3–4 bpm). Caudal to these segments stimulation in trial experiments failed to get effects on any recorded functional parameter.

The left and right sympathetic chains were stimulated in random order. The sequence in which the different segmental levels were stimulated was also random. All measurements were made during a steady state at baseline and when the new steady state heart rate was reached during nerve stimulation.

### 2.6 Data recording and statistical analysis

The Powerlab 16/30 system (AD Instruments Ltd., Chalgrove, UK) was used to record the signals, which were processed at 2 kHz. Data are mean ± SEM; compared using ANOVA or paired *t*-test for which statistical significance was taken at 5% level (*p* < 0.05).

## 3 Results

### 3.1 Effect of left or right sympathetic chain stimulation on heart rate (HR) and left ventricular pressure (LVP)

At each electrode pairing, from T4-T5 to T1-T2, both the right sympathetic and left sympathetic chain augmented HR and LVP (see Fig. 1 in [20]). The effects were larger at successive rostral levels as more sympathetic preganglionic axons were activated. The effects of stimulation of the right chain on HR were greater than left chain stimulation. In contrast the left sympathetic chain elicited larger LVP responses during constant pacing, than did stimulation of the right sympathetic chain. Notably, although stimulation at T5-T6 on the left side increased both HR and LVP, the right side stimulation at this level was ineffective.

#### 3.1.1 Chronotropic effects

At T5-T6 the left sympathetic chain increased HR to 136.5 ± 4.6 bpm (11.2 ± 6.1% change from baseline). Thereafter comparing the mean increases in HR from left with those from right sympathetic stimulation at successive levels, the response to left sympathetic stimulation at T4-T5 was 145.4 ± 3.9 bpm (15.1 ± 2.7%), and to right sympathetic stimulation 167.6 ± 11.5 bpm (25.9 ± 7.5%); T3-T4, left sympathetic stimulation increased HR to 155.0 ± 6.5 bpm (19.0 ± 4.4%), right sympathetic to 170.4 ± 7.0 bpm (29.0 ± 4.3%); T2-T3, left increased to 171.3 ± 5.3 bpm (29.4 ± 4.6%) and right to 189.5 ± 5.7 bpm (43.6 ± 4.7%); T1-T2, left increased to 181.8 ± 7.6 bpm (41.1 ± 5.6%) and right sympathetic to 211.8 ± 6.9 bpm (59.9 ± 6.0%).

#### 3.1.2 Inotropic effects

At T5-T6 the LVP, measured during constant ventricular pacing, increased to 38.8 ± 6.1 mm Hg (13.0 ± 2.1% from baseline) on stimulating the left sympathetic chain but there was no effect of stimulating the right. At each of the succeeding segments left sympathetic chain stimulation elicited larger increases in LVP compared to right stimulation as follows: T4-T5, left side effect was 40.5 ± 4.0 mm Hg (17.4 ± 3.0%), right was 36.8 ± 4.0 mm Hg (5.6 ± 1.5%); T3-T4, left was 43.6 ± 4.0 mm Hg (20.9 ± 2.6%), right was 38.6 ± 4.0 mm Hg (7.0 ± 1.6%); T2-T3 left was 45.3 ± 3.5 mm Hg (28.8 ± 2.4%), right was 38.5 ± 3.4 mm Hg (15.1 ± 2.3%); T1-T2 left was 51.3 ± 3.9 mm Hg (40.3 ± 5.4%), right was 40.7 ± 3.7 mm Hg (20.7 ± 3.2%). At all levels the left sympathetic chain was dominant and T1-T2 caused the largest increase.

### 3.2 Effect on ventriculo-atrial (VA) conduction

Dromotropic effects were measured during constant pacing with left and right sympathetic chain stimulation at each electrode pairing between T1-T6 (see Fig. 2 in [20]). A reduction in VA conduction was caused by both left and right sympathetic chain stimulation but the effects were largest following left stimulation. At T5-T6 left sympathetic stimulation decreased VA conduction from 143.7 ± 1.8 ms to 137 ± 1.7 ms (−4.6 ± 0.2%). There was no effect of right stimulation at this level. At T4-T5 and T3-T4 stimulation, both left and right sides elicited similar reductions to T5-T6 on the left side. More rostral the reductions were larger so at T2-T3 left side was 130.1 ± 6.7 ms (−12 ± 5.0%) and right was less at 137.2 ± 3.5 ms (−6.4 ± 1.7%). The reduction in VA conduction was largest at T1-T2 where left stimulation caused a change to 125.6 ± 6.6 ms (−15.5 ± 0.2%) whereas the right side was 135.6 ± 1.9 ms (−6.8 ± 1.1%).

### 3.3 Monophasic action potential (MAP) changes

Changes in MAP were measured from the anterior surface of the left ventricle during constant ventricular pacing.

#### 3.3.1 Effect on MAP duration

**Fig. 2:**
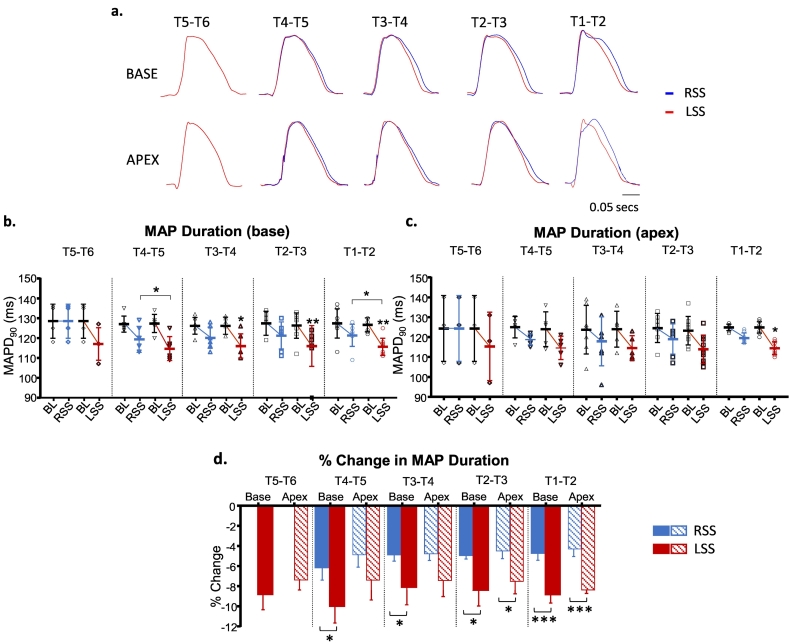
Effects of right and left chain stimulation on monophasic action potential (MAP) duration. (a) Basal and apical MAP traces during right sympathetic stimulation (RSS) and left sympathetic stimulation (LSS) at the levels stimulated between T1-T6. (b) Basal MAP duration during baseline (BL) and RSS and LSS between T1-T6. (c) Apical MAP duration during BL and RSS and LSS between T1-T6. (d) Percentage change in MAP duration at the base and apex with RSS and LSS. Data represent mean ± SEM. *P < 0.05, **P < 0.01, ***P < 0.001.

The effects of left and right sympathetic chain stimulation on the MAP duration were measured at electrode pairs between T1-T6 at the LV base and LV apex. Both left and right sympathetic stimulation caused reductions in MAP duration. The largest changes were induced by the left sympathetic neurones. At T5-T6, left sympathetic stimulation decreased MAP duration from 128.2 ± 4.3 ms to 117.0 ± 4.1 ms, a change of 11.2 ms (−8.7 ± 1.6%) at the base and from 124.3 ± 9.6 ms to 115.3 ± 9.9 ms a change of 9.0 ms (−7.4 ± 0.9%) at the apex (Fig. 2). Thereafter the decreases in MAP duration were similar to T5-T6 for base and apex. On stimulating the right sympathetic chain at T5-T6 there was no effect on MAP duration, at base or apex. Right sympathetic stimulation induced a small but significant decrease of −6.1 ± 1.3% base and at apex −4.9 ± 1.2% first at T4-T5 and this was little changed by stimulation at subsequent sites. When comparing base and apex data as shown in Fig. 2d, left sympathetic stimulation showed a greater change in MAP duration at the base although the difference is small. This effect is particularly evident at the caudal segments.

#### 3.3.2 Effect on effective refractory period (ERP)

Left sympathetic stimulation at T5-T6 decreased the ERP from 147.5 ± 1.4 ms to 131.7 ± 1.7 ms, a change of −8.1 ± 4.4% (see Fig. 3 in [20]) but there was no effect of right sympathetic at this level. At subsequent levels the ERP was further shortened by stimulation of each sympathetic chain. At T4-T5 on the left the ERP reduction was −7.7 ± 1.8%; right at T4-T5 was 3.6 ± 1.3%; T3-T4 left was −11.0 ± 1.9%, right was −9.6 ± 1.7%; T2-T3 left was −12.6 ± 2.5%, right was −9.9 ± 1.8%. T1-T2 stimulation on the left side caused a much larger reduction in ERP to 121.5 ± 2.6 ms (−17.6 ± 2.0%). A similar enhancement was not observed for right side stimulation where the reduction was −11.3 ± 1.7%, not significantly different to the previous segment.

### 3.4 Effect of left and right sympathetic chain stimulation on electrical restitution of MAP duration

**Fig. 3:**
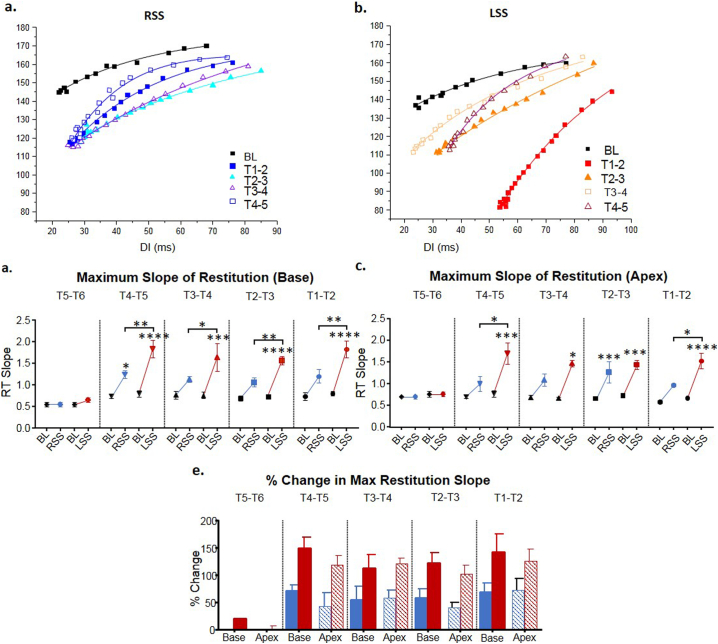
Effect of right and left sympathetic stimulation on maximum slope of restitution. Restitution slopes at the levels stimulation between T1-T6 during (a) RSS and (b) LSS with exponential curve fit (MAPD_90_ = maximum MAPD_90_ [1 − e^−DI/T^]). (c) Maximum slope of restitution at the base from baseline (BL) to right sympathetic stimulation (RSS) and BL to left sympathetic stimulation (LSS) at the levels stimulated between T1-T6. (d) Maximum slope of restitution at the apex from BL to RSS and BL to LSS at the levels stimulated between T1-T6. (e) Percentage change in the maximum slope of restitution at the base and apex for RSS and LSS at spinal segments T1-T6. Data represent mean ± SEM. *P < 0.05, **P < 0.01, ***P < 0.001, ****P < 0.0001.

Restitution curves of the MAP duration at corresponding diastolic intervals were plotted, during baseline, left sympathetic stimulation and right sympathetic stimulation (see Fig. 4 in [20]). At T5-T6, there was a small effect of left sympathetic stimulation, which steepened the maximum slope of restitution by 18.3 ± 1.6% at the base but had no effect at apex (1.4 ± 6.1%) (Fig. 3). There was no significant effect of right stimulation at base or apex at this level. For the remaining segments, left sympathetic stimulation steepened the maximum slope of restitution at the base to a greater degree than right stimulation. At the apex, left sympathetic stimulation also steepened the maximum slope of restitution more than right.

Stimulation of the left sympathetic chain at segment T4-T5, for both base (left was 147.8 ± 21.7% increase, right was 69.9 ± 11.3%) and apex (left was 118.2 ± 21.4% increase, right was 43.0 ± 24.0%), was more effective in increasing the slope of restitution, since there was little additional change during stimulation of successive rostral segments. At most levels, left stimulation was shown to have a dominant effect at increasing maximum slope of restitution at the base in comparison to apex (Fig. 3e). This was more apparent at caudal levels.

### 3.5 Effect of left and right sympathetic chain stimulation on VF threshold (VFT)

**Fig. 4:**
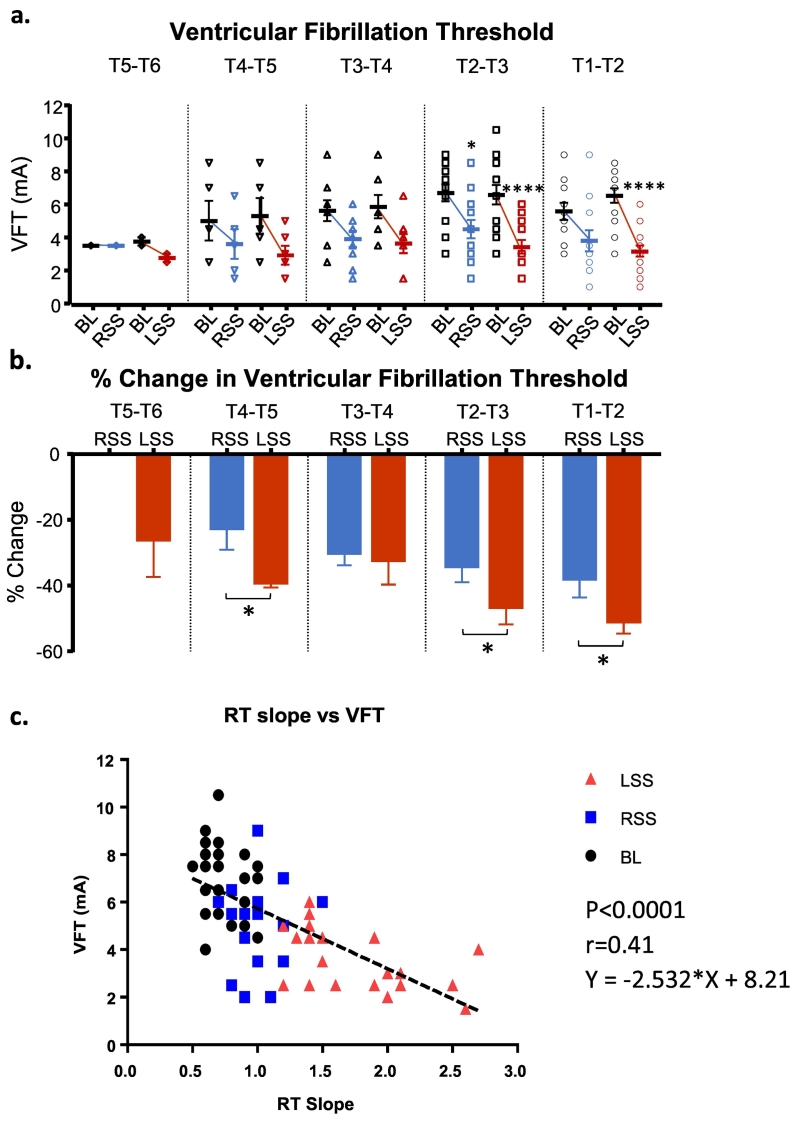
Effect of right and left sympathetic stimulation on ventricular fibrillation threshold (VFT) (a) VFT changes from baseline (BL) to right sympathetic stimulation (RSS) and BL to left sympathetic stimulation (LSS) at the levels stimulated between spinal segments T1-T6. (b) Percentage change in VFT for RSS and LSS at spinal segments T1-T6. Data represent mean ± SEM, *P < 0.05, ****P < 0.0001. (c) Relationship between maximum slope of restitution and VF threshold with linear regression analysis. Individual symbols represent values obtained at BL, LSS and RSS. Equation of line is y = 2.532 ∗ X + 8.21, *P* < 0.001.

As shown in Fig. 4 the left sympathetic chain stimulation elicited larger decreases in VFT compared to right stimulation at each segment. Significantly at T5-T6, VFT was unchanged by right stimulation but was decreased by left stimulation to 2.8 ± 0.3 mA (−25.9 ± 11.6%). At each subsequent segment both left and right stimulation caused a decrease. The largest additional changes in VFT occurred between segments T4-T5 at which left was 2.9 ± 0.6 mA (−39.0 ± 1.7%) and right was 3.6 ± 0.9 mA (−22.3 ± 6.8%).

### 3.6 Correlation between sympathetic modulation of maximum slope of restitution and VFT

In Fig. 4c, maximum slope of restitution is plotted against the corresponding VFT. There is a significant relationship between the two parameters. The graph shows the more right shifted effects of left sympathetic neurones and indicates that they have a greater influence on the stability of rhythm than right sympathetic stimulation.

## 4 Discussion

The cardiac sympathetic nerve input from the spinal cord via the thoracic ganglia into the heart involves a complex arrangement of neuronal connections with the resultant sophisticated control of function (reviewed in [21]). It has long been established that functionally distinct populations of target specific neurones in the mammalian superior cervical ganglion are innervated by preganglionic neurones arising from different levels of the spinal cord [22]. Using a novel refinement of the original isolated innervated heart preparation that allows controlled segmental stimulation, the findings of the present study show that the stellate ganglia supplying the heart are similarly organized. The results of the present experiments indicate that there are groups of preganglionic neurones in different spinal segments that selectively target functionally distinct cardiac postganglionic neurones in the stellate ganglia. It is also shown there is a right-left difference in the strength of the functional effects.

The interpretation of the data from the method employed, by which additional preganglionic axons joining the sympathetic chain at each segment were stimulated, seems justified by studies of the electrophysiological properties of sympathetic ganglia. Larrabee and Bronk [10] and Birks et al. [23] examined the electrophysiological properties of postganglionic neurones in the cat stellate ganglion and clearly showed that maximal stimulation of different populations of preganglionic axons in the sympathetic chain, evoked compound action potentials in different groups of postganglionic neurones in the inferior cardiac nerve. Therefore, in the present study it seems reasonable to conclude that the magnitude of the functional cardiac response reflected the number of preganglionic axons activated at each electrode position. This interpretation accords with evidence that postganglionic neurones are organized into single functional pathways and individual sympathetic preganglionic neurones have a dominant influence over only one such neurone [7,9,12,24,25]. One should bear in mind, however, there are significant inter-neuronal connections at the paravertebral and intrinsic cardiac nervous system which adds complexity to the understanding of the precise mechanistic pathways mediating important cardiac functions [[26], [27], [28]].

In the present study, as we stimulate electrode pairs from caudal to rostral, more axons are stimulated resulting in a progressive increase in the size of the cardiac response. This is seen for chronotropic and inotropic responses, where there is an additional increase in HR or LVP at each pair of electrodes from the caudal pair to the most rostral electrodes. This was not as a result of simple summation as the size of the response at T1-T2 was less than the sum of the sequence of increases from T5-T6. However, the size of the compound action potential elicited in the cardiac postganglionic nerves reflects the number of postganglionic neurones activated [29]. Therefore, the data suggest that sympathetic terminals from each spinal segment converge onto groups of neurones in a single functional pool of postganglionic neurones in the stellate ganglion a characteristic of all autonomic ganglia [22]. It is also clear from this data that the cardiac acceleratory preganglionic neurones are more abundant on the right side of the spinal cord whereas the inotropic neurones are better represented on the left side, in accord with previous studies [2,16,18]. The largest additional increase for each function is from T1-T3 implying that within these segments is located the largest proportion of spinal chronotropic sympathetic neurones on the right side or inotropic neurones on the left side. Such an arrangement of functional heterogeneity between right and left cardiac sympathetic innervation has long been indicated [30,31] and was recently expanded by a quantitative study in our lab [17]. The present results describe a more extensive examination to determine if cardiac sympathetic preganglionic neurones are selectively represented in more widespread spinal segments, an idea first tested in Bob Wurster's lab in anesthetized open chest dogs [13]. In the latter study electrical stimulation of each of the ventral roots from T1 to T5 on both left and right sides showed that stimulation of T2 was the most effective in increasing HR, and LVP at several quite discrete areas on the anterior surface of the right and left ventricles. In the present studies using the innervated isolated rabbit heart, we have extended these findings. This preparation enables the effects of nerve stimulation to be tested without confounding effects of spinal reflexes or changes in haemodynamic loads or circulating hormones. We have also previously demonstrated novel data showing the effects of nerve stimulation on ventricular repolarisation gradient, which were different from perfusing with neurotransmitter analogues [32]. The current results showed there was a successive graded increase in ventricular contractility from T5-T6 to T1-T2 and the largest additional effect was from T1-T2 on the left side, suggesting that T1 spinal segment has a dominant influence on LVP. These data give a different picture of sympathetic influence on LVP than do the results reported by Norris et al. [13] on dogs which showed that the degree of positive inotropism measured at five sites on the anterior surface of the left ventricle was on average similar for both left and right ventral root stimulation. In our study, in which the cavity pressure and hence force of contraction of the whole left ventricle was measured, the effect of the left sympathetic nerves was virtually double the effect of right sympathetic nerves at every level. Furthermore, there were no chronotropic or inotropic effects when stimulating the T5-T6 on the right side, suggesting the outflow at this level on the left side is selectively cardiac.

With regard to electrophysiological effects, a more dominant left sympathetic action was observed. Firstly, the effect of stimulation in reducing MAP duration was evident both at base and apex on the anterior ventricle surface, and the left side effects were greater compared to right, an effect we have shown previously [17]. However, in the latter experiments, stimulation was restricted to sympathetic stimulation at the T2-T3 level. In the present study, by stimulating the sympathetic chain at different sites from T6-T1, the results go further in showing the effect on MAP duration is mainly an action of preganglionic neurones located in the most caudal spinal segments on the left side. A previous study using optical mapping of the innervated isolated rabbit heart showed a differential gradient of base to apex innervation of sympathetic nerves with bilateral sympathetic stimulation [32]. Here we have shown, although there was a trend for a greater change in MAP duration at the base in comparison to apex, these effects were small. Nonetheless, these results provide evidence of spatial heterogeneities of sympathetic effects and indicate that there may be a greater innervation of sympathetic fibers and a higher density of IK_s_ channels at the base of the ventricle. The differences observed between base and apex were particularly evident at T4-T5 for both MAP duration and maximum slope of restitution suggesting regional innervation and preferential supply to the base at caudal levels. This merits further investigation into the regional differences in sympathetic innervation of the left ventricle.

These changes were also accompanied by reductions in the ERP. The left sympathetic effects on ERP were greater than those elicited by right sympathetics and for each, the response from caudal thoracic segments was remarkably large considering the relatively small HR changes which at these levels. In contrast, at T1-T2 the reduction in ERP was larger, and this might partly be explained by the greater effects on HR at this level. However, this would not appear to conform to the change in the HR and ERP on the right side. Left side preganglionic neurones had a clear predilection over right neurones in shortening the ERP even though right sympathetics had a dominant HR effect. An explanation may lie in the greater APD changes evoked by the left sympathetics and their denser innervation of the left ventricle. These data contrast with the more general observations by Yanowitz et al. [33] that suggested that left sympathetic activity had less influence than right based on T wave changes on the ECG.

Significantly, our study has revealed that in the rabbit, the caudal spinal segments contain a group of sympathetic preganglionic neurones that have a major influence on electrical stability of the heart and a high potential to cause abnormal cardiac rhythms. They selectively activate cardiac postganglionic neurones, which reduce APD, and strongly influence restitution and VFT. APD restitution is an important predictor of arrhythmogenesis. The restitution hypothesis states that a steep APD restitution curve with the slope of the curve >1, means that small changes in diastolic interval would lead to large changes in APD and hence wavebreaks can occur which facilitate fibrillation [34]. We have previously shown that sympathetic stimulation increased the slope of APD restitution which led to increased electrical alternans and susceptibility to VF [19].

Functional consequence of cardiotopic innervation of the sympathetic nerves on regional electrophysiology requires detailed knowledge of innervation, ion channel distribution and dynamic behaviour of the heart. This merits further investigation and would require mechanistic insight on regional APD changes in both activation and repolarisation i.e. with optical mapping, which is a line of investigation our laboratory is pursuing with this refined preparation. Also, important processes such as Ca^2+^ handling are under significant influence from sympathetic stimulation [35] and their contribution towards increased arrhythmogenicity with regional sympathetic stimulation deserves detailed investigation in both normal hearts and in disease [36].

### 4.1 Clinical implications

The removal or pharmacological block of the stellate ganglion is a major clinical procedure to treat long QT syndrome and a variety of cardiac arrhythmias. Early work had suggested that left cardiac sympathetic denervation (LCSD) is favoured over right CSD as it has minimal effects on heart rate, together with paradoxical arrhythmogenic effects from right CSD whereas LCSD protects against VF [37]. This is in accord with the data provided in our current studies showing a predilection of right sympathetic chain stimulation towards heart rate effects whilst left sympathetic stimulation has predominant effects on ventricular electrophysiology and VF initiation. LCSD involves an incision at the base of the neck and surgical excision of the left stellate ganglion, together with the thoracic ganglia (T1 to T4). With LCSD, the upper half of the stellate ganglion is preserved to prevent incidence of Horner's syndrome [38]. The resulting reduction in catecholamine release gives antiarrhythmic effects without causing post-denervation supersensitivity [39] with supportive evidence for such mechanisms in arrhythmic syndromes [40]. Whilst such treatment has met with impressive success with recent clinical evidence supportive of left and bilateral CSD [[40], [41], [42], [43], [44]], it is not without serious side effects due to it also removing the sympathetic supply to the forelimbs and head causing palmar and facial anhydrosis, loss of vasomotion, and poorer visual and salivary control [45]. Clearly a more focused treatment that targets the responsible sympathetic preganglionic neurones that are excessively active would be preferable. This has been investigated recently by Buckely et al. [46] in in vivo porcine studies in relation to the concept that cranial and caudal input into the stellate ganglia provide differential effects on the heart. They showed that after removal of the caudal stellate ganglia to T2 level, T3 stimulation no longer produced changes in ventricular activation recovery intervals [49], suggesting that T1-T2 excision would suffice if targeting arrhythmic effects on the heart. The results of our current study extends this concept to show that the predominant electrophysiological effects and those of VF initiation were concentrated at a more focussed region in the rabbit at the T4-T6 sympathetic outflow on the left side. This would propose an alternative, more targeted approach to present practice, i.e. allow the removal of the outflow that has a potential to cause arrhythmia whilst mitigating the collateral side effects of wide denervation but the same time preserving important sympathetic nerve function. It is appreciated that this may be surgically challenging but our study suggests it is an approach worth exploring further in humans.

### 4.2 Limitations

We consider the methodology used in this study justified because of the well-documented knowledge of the electrophysiology of preganglionic synapses in thoracic ganglia [19,47]. The effects of subliminal fringe, spatial, temporal facilitation, occlusion, convergence and divergence, were ameliorated by supramaximal stimulation at each electrode position. We accept that the electrophysiology tests were limited to the anterior surface of the heart but stellate stimulation in pigs and humans suggests that similar actions are likely to have occurred on the posterior aspect of the left ventricle [[48], [49], [50]]. In using the rabbit as our experimental model we are aware that extrapolation to other animals including humans has to be done with caution. Nonetheless there is plenty of evidence for similarity in the anatomy and function of the autonomic innervation of all mammals studied so far [22]. We realise that the isolation from confounding physiological factors represented by our innervated heart preparation may represent an over-simplification of the basic control mechanisms in intact animals and humans, in which preganglionic neurone activity would be influenced by background activity and autonomic tone. However, in this study we are assessing the efferent outflow in the absence of confounding factors and it would be surprising if the functional anatomy is different to what we describe in the rabbit.

### 4.3 Conclusions

Our results reveal previously uncharacterised properties of the preganglionic innervation of cardiac neurones in the stellate ganglion of rabbits. The results draw attention to a significant role for preganglionic neurones in the lower thoracic spinal outflow (T4-T6) on the left side having a dominant action over cardiac ventricular electrophysiology and hence arrhythmogenic potential.

## Conflicts of interest

None.
